# Standardised spider (Arachnida, Araneae) inventory of Lammi, Finland

**DOI:** 10.3897/BDJ.8.e50775

**Published:** 2020-03-13

**Authors:** Arttu Soukainen, Timo Pajunen, Tuuli Korhonen, Joni Saarinen, Filipe Chichorro, Sonja Jalonen, Niina Kiljunen, Nelli Koskivirta, Jaakko Kuurne, Saija Leinonen, Tero Salonen, Veikko Yrjölä, Caroline Fukushima, Pedro Cardoso

**Affiliations:** 1 Faculty of Biological and Environmental Sciences, University of Helsinki, Helsinki, Finland Faculty of Biological and Environmental Sciences, University of Helsinki Helsinki Finland; 2 Laboratory for Integrative Biodiversity Research (LIBRe), Finnish Museum of Natural History (LUOMUS), University of Helsinki, Helsinki, Finland Laboratory for Integrative Biodiversity Research (LIBRe), Finnish Museum of Natural History (LUOMUS), University of Helsinki Helsinki Finland; 3 Finnish Museum of Natural History (LUOMUS), University of Helsinki, Helsinki, Finland Finnish Museum of Natural History (LUOMUS), University of Helsinki Helsinki Finland; 4 Department of Biological and Environmental Science, University of Jyväskylä, Jyväskylä, Finland Department of Biological and Environmental Science, University of Jyväskylä Jyväskylä Finland

**Keywords:** Arthropoda, boreal forest, COBRA, sampling

## Abstract

**Background:**

In June 2019, an ecology field course of the University of Helsinki was held at Lammi Biological Station, Southern Finland. Within this course, the students familiarised themselves with field work and identification of spiders and explored the diversity of species in the area. Three sampling plots were chosen, one in grassland and two in boreal forest, to demonstrate the sampling techniques and, by applying a standardised protocol (COBRA), contribute to a global spider biodiversity project.

**New information:**

The collected samples contained a total of 3445 spiders, of which 1956 (57%) were adult. Only adult spiders were accounted for in the inventory due to the impossibility of identification of juveniles. A total of 115 species belonging to 17 families were identified, of which the majority (58 species, 50%) were Linyphiidae. Lycosidae and Theridiidae both had 11 species (10%) and all the other families had seven or fewer species. Linyphiidae were also dominant in terms of adult individuals captured, with 756 (39%), followed by 705 (36%) Lycosidae. Other families with more than 100 individuals were Thomisidae (196, 10%) and Tetragnathidae (102, 5%). The most abundant species were the lycosids *Pardosa fulvipes* (362, 19%) and *Pardosa riparia* (290, 15%) and the linyphiid *Neriene peltata* (123, 6%).

## Introduction

Finland's dominant biome is the taiga, where swamps and lakes are common within large forest expanses. The area is part of a transition zone between the northernmost coniferous forests and the southernmost deciduous forests. The Finnish flora and fauna are some of the best known in the world, due to the tradition of taxonomic work and low diversity in species. The fauna of Finland is relatively new, as it is only about 10,000 years from the end of the last Glacial Maximum. During that time, the area was completely covered with ice. As a result, most of the organisms have migrated from the South during the last thousands of years and this process is still ongoing. There are hardly any endemic species with only few exceptions. About 45000 multicellular species are currently known in the region and Finland is currently the only country in the world where threat level for species have been extensively assessed three times in accordance with the International Union for the Conservation of Nature (IUCN) criteria (Rassi et al. 2000, Rassi et al. 2010, Hyvärinen et al. 2019).

The traditions of Finnish faunistic work are strong. In the 18th century, Finland was part of Sweden and, at that time, natural scientists had good contacts with Carl von Linné. For spiders in particular, Seppo Koponen described the history of Finnish arachnology in 2010 (Koponen 2010). The first spider list was compiled by A. von Nordmann in 1863 and it contained 140 species (Nordmann 1863). This list was later supplemented by F. W. Mäklin, K. E. Odenwall and T. H. Järvi and, by the beginning of the 20th century, the list already contained 255 species (Mäklin 1874, Järvi 1906, Odenwall and Järvi 1901). Pontus Palmgren, the most prominent Finnish arachnologist of the century, began a long-term study in the Tvärminne area, reporting 425 species from that area alone. Even today, Tvärminne's spider fauna is one of the best known in the world. The most recent addition to the area is a data paper from 2017 (Cardoso et al. 2017). One of Palmgren's main work was 'Die spinnenfauna Finlands und Ostfennoskandiens' I-VIII (Palmgren 1939, Palmgren 1943, Palmgren 1950, Palmgren 1974a, Palmgren 1974b, Palmgren 1975, Palmgren 1976, Palmgren 1977). This spider identification book series is still an important tool for researchers today and was also an important key in the identification process of this paper. In addition to Palmgren, Pekka Lehtinen (Lehtinen 1964, Lehtinen et al. 1979), Seppo Koponen (Koponen 1977, Koponen et al. 2007), Timo Pajunen (Pajunen et al. 2009, Pajunen and Väisänen 2015) and Niclas Fritzén (Fritzén 2002, Fritzén 2005, Fritzén 2012, Fritzén et al. 2015) have been other significant contributors to the faunistic work of Finnish spiders. Today, the Finnish spider list contains 647 species (Koponen et al. 2016), of which 21 are threatened and 67 near threatened (Hyvärinen et al. 2019).

Established in 1953, the Lammi Biological Station is surrounded by diverse lakes, forests, streams, marshes and ridges. Although there have been numerous spider courses held at the station over the years, no comprehensive list of spiders has been compiled from the area. Thus, this data paper is the first faunistic spider-related publication in the region.

In the spring/summer of 2019, the station hosted an ecology field course at BSc level, during which it was possible to sample the three plots in a standardised way. One of the sampling goals was to collect high-quality data for a global spider biodiversity project (http://biodiversityresearch.org/research/biogeography). Thanks to this sampling protocol , the data produced can be compared to results obtained in many other areas around the world.

## Sampling methods

### Study extent

**Figure 1.**
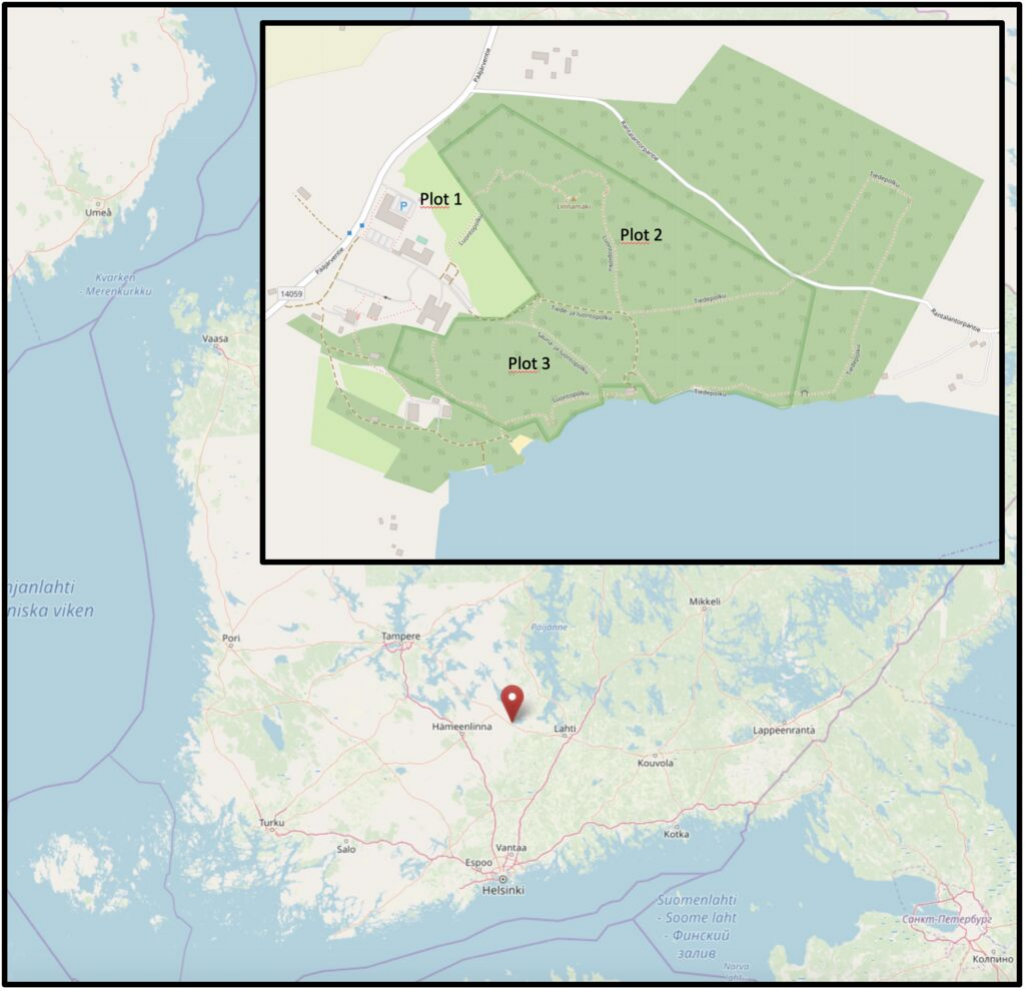
Location of the three sampled plots in southern Finland (data from OpenStreetMaps).

**Figure 2.**
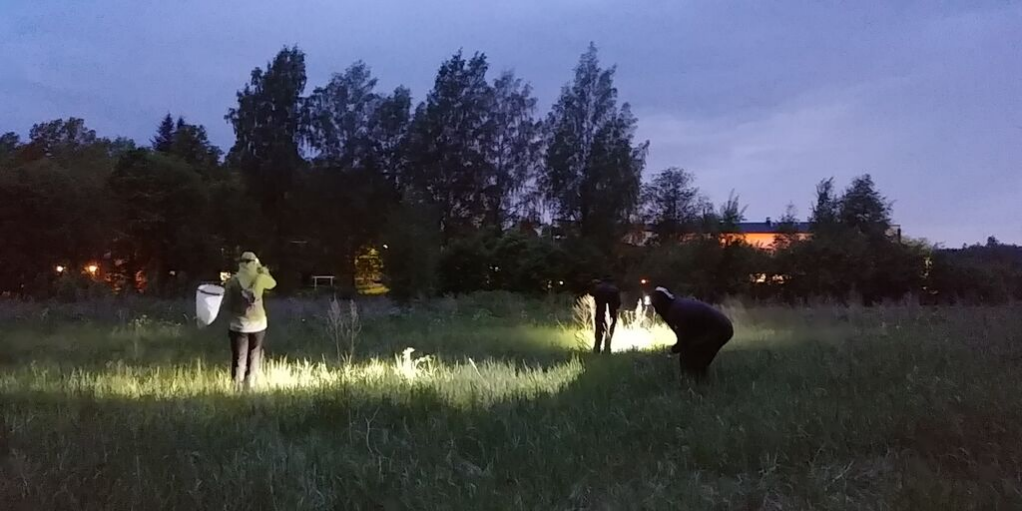
Night sampling at Plot 1 in grassland (photo by Pedro Cardoso).

**Figure 3.**
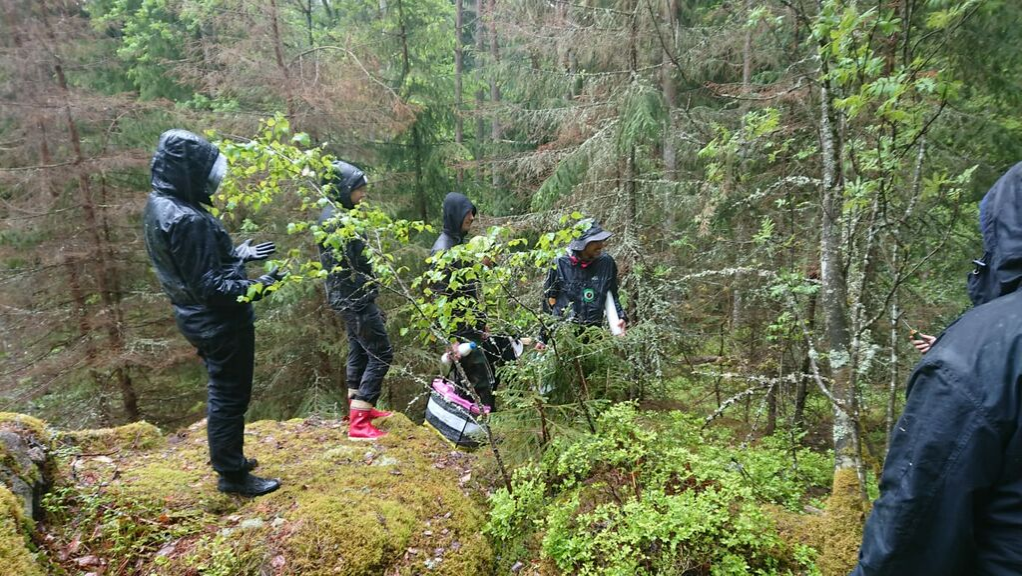
Setting up Plot 2 in forest (photo by Sonja Jalonen).

Three 50 × 50 m plots near the biological station were selected for sampling (Fig. 1; Table 1). The plots were selected to maximise species coverage around the station. Plot 1 was located in a field on the edge of a forest where a variety of grasses, such as *Deschampsia cespitosa* and *Calamagrostis arundinacea* grew (Fig. 2). Plot 2 was located in a forest dominated by spruce (*Picea abies*) and pine (*Pinus sylvestris*) and where the bottom of the forest was rich in moss (Fig. 3). Plot 3 was located in a dense deciduous forest with many species of trees such as birch (*Betula* sp.), aspen (*Populus tremula*), maple (*Acer platanoides*) and alder (*Alnus incana*). The field layer was flowering and lush, with the most common shrubs being mountain currant (*Ribes alpinum*) and common hazel (*Corylus avellana*).

### Sampling description

COBRA - Conservation Oriented Biodiversity Rapid Assessment - was used to collect samples from the three different plots selected. We made a total of 24 hours of active sampling per plot. In forest habitats, this includes aerial night sampling (4 hours/plot), day/night sweeping (2 hours/plot each), day/night beating (2 hours/plot each) and pitfall traps (48 traps distributed for 12 samples). The methods for the grassland were the same, except beating was replaced by sweeping (total of 4 hours/plot day and night) and aerial night sampling was replaced by ground night sampling. This protocol was first proposed for Mediterranean spiders (Cardoso 2009) and has been later adapted to apply in the tropics (Malumbres-Olarte et al. 2016) and islands (Emerson et al. 2016). This publication follows a similar data paper previously made for Hanko, Finland (Cardoso et al. 2017).

**Study dates**: The samples were collected during May/June 2019. Pitfall traps were left in the field on 25th of May and collected 10th of June. All other, active, methods were conducted on the 4th, 5th and 6th of June.

## Geographic coverage

### Description

Lammi, Finland

### Coordinates

61.05 and 61.06 Latitude; 25.04 and 25.05 Longitude.

## Taxonomic coverage

### Taxa included

**Table:** 

Rank	Scientific Name	Common Name
order	Araneae	Spiders

## Temporal coverage

**Data range:** 2019-5-25 – 2019-6-10.

## Usage rights

### Use license

Open Data Commons Attribution License

## Data resources

### Data package title

COBRA_Finland_Lammi

### Resource link

https://doi.org/10.15468/kauh71

### Number of data sets

1

### Data set 1.

#### Data set name

COBRA_Finland_Lammi

#### Number of columns

21

#### 

**Data set 1.** 

Column label	Column description
occurrenceID	An identifier for the Occurrence (as opposed to a particular digital record of the occurrence).
basisOfRecord	The specific nature of the data record.
recordedBy	A list (concatenated and separated) of names of people, groups or organisations responsible for recording the original Occurrence.
individualCount	The number of individuals represented present at the time of the Occurrence.
lifeStage	The age class or life stage of the biological individual(s) at the time the Occurrence was recorded.
samplingProtocol	The name of, reference to, or description of the method or protocol used during an Event.
eventRemarks	Comments or notes about the Event.
eventDate	The date-time or interval during which an Event occurred.
locationID	An identifier for the set of location information (data associated with dcterms:Location).
country	The name of the country or major administrative unit in which the Location occurs.
county	The full, unabbreviated name of the next smaller administrative region than stateProvince (county, shire, department etc.) in which the Location occurs.
locality	The specific description of the place.
minimumElevationInMeters	The lower limit of the range of elevation (altitude, usually above sea level), in metres.
maximumElevationInMeters	The upper limit of the range of elevation (altitude, usually above sea level), in metres.
decimalLatitude	The geographic latitude (in decimal degrees, using the spatial reference system given in geodeticDatum) of the geographic centre of a Location.
decimalLongitude	The geographic longitude (in decimal degrees, using the spatial reference system given in geodeticDatum) of the geographic centre of a Location.
geodeticDatum	The ellipsoid, geodetic datum or spatial reference system (SRS) upon which the geographic coordinates given in decimalLatitude and decimalLongitude are based.
identifiedBy	A list (concatenated and separated) of names of people, groups or organisations who assigned the Taxon to the subject.
dateIdentified	The date on which the subject was identified as representing the Taxon.
scientificName	The full scientific name, with authorship and date information, if known.
taxonRank	The taxonomic rank of the most specific name in the scientificName.

## Additional information

A total of 3445 spiders, of which 1956 (57%) adults, were collected (Table 2; Cardoso 2020). These belonged to 115 species (Table 2) and voucher specimens are deposited at Luomus - the Finnish Museum of Natural History). Of these, 58 species (50%) were Linyphiidae, 11 (10%) Theridiidae and 11 (10%) Lycosidae. All other families had seven or less species represented. Linyphiidae were also dominant in terms of adult individuals captured, with 756 (39%), followed by 705 (36%) Lycosidae, 196 (10%) Thomisidae and 102 (5%) Tetragnathidae. All other families had less than 100 individuals. The most abundant species were *Pardosa fulvipes* (362), *Pardosa riparia* (290) and *Neriene peltata* (123). Only these species had more than 100 individuals captured. Plot 1 had the most species (78), 68% of all species captured, followed by Plot 3 (45 species, 39%) and finally Plot 2 (34 species, 30%).

### Discussion

Most species in the inventory are common and widespread in Finland, with the exception of *Diaea dorsata*, a Thomisidae, of which only one juvenile was captured and *Clubiona diversa*. *Diaea dorsata* was previously considered threatened in Finland (Rassi et al. 2010), but in the latest threat assessment, it has been classified as least concern (Hyvärinen et al. 2019). This species was previously found on the southernmost coast of Finland and the Åland Islands (an archipelago in the Baltic Sea), but has since spread to the north (http://biolcoll.utu.fi/arach/aran2013/Diaedors.pdf). The presently collected *Diaea dorsata* samples are the northernmost observation of this species in Finland. *Clubiona diversa* is found in the southern parts of the country and collected samples for this species are in the northernmost parts (http://biolcoll.utu.fi/arach/aran2013/Clubdive.pdf). Global warming is the most likely cause of the northwards movement of many invertebrates (Parmesan et al. 1999), including spiders in Finland. Many Finnish spider species are known to be spreading further north (Fritzén et al. 2015) and these two species are probably examples of such change.

Another interesting case is for *Dicymbium nigrum.* Our specimens belong to the subspecies *D. nigrum brevisetosum* Locket, 1962. This subspecies has not been reported from Finland before, so it is an addition to the region. It is possible that the subspecies is not on the Finnish spider list simply due to the fact that it has been incompletely identified in the past.

Compared with similarly-sampled forest sites in the southern coast of Finland (Cardoso et al. 2017), where four sites had between 56 and 62 species, the forest areas in Lammi had fewer species. The southern coast of the country is known to be a hotspot for Finnish fauna, namely spiders (Cardoso et al. 2017) and these results are not surprising, even if contrasting.

The fact that the grassland in Plot 1 was the richest might be due to its location within a mosaic of different habitat types, namely forest and urban areas. The spillover of vagrant species, typical from bordering habitats, has contributed to the richness in this particular area. Amongst forest areas, further away from other habitat types and therefore mostly free from border effects, Plot 3 had the most complex structure and richest plant diversity, explaining its higher richness compared with the more homogeneous Plot 2. The number of adult individuals captured was also significantly larger in Plot 1 (1307) compared to Plot 2 (214) and Plot 3 (435). It is noteworthy that most of the individuals in Plot 1 were collected with pitfall traps (1098, 84%), which also contributes to the large differences in richness. Open grasslands favour actively-moving species which operate at ground level. For example, *Pardosa fulvipes* and *Pardosa riparia* are ground hunters and were very numerous in our traps. It should also be noted that, during the collection of the samples, the weather was sunny and warm for several days. The pitfall trap cover (which prevents debris from dripping into the pit) creates a shady spot that can attract spiders that seek protection from direct sunlight in such an open environment. In forest habitats, this is not as much of an issue.

## Figures and Tables

**Table 1.** Coordinates of sampling plots. The plots average 123 metres above sea level.

**Plot**	**Habitat**	**Latitude**	**Longitude**	**Metres above sea level**
1	Grassland	61.055564	25.041543	110-120
2	Forest	61.054843	25.047673	120-130
3	Forest	61.052627	25.043498	100-110

**Table 2.** Richness and abundance of species per plot (adults only).

Family	Species	Plot 1	Plot 2	Plot 3	Total
Araneidae	*Araneus sturmi* (Hahn, 1831)	1			1
Araneidae	*Cyclosa conica* (Pallas, 1772)		7		7
Clubionidae	*Clubiona diversa* O. P.-Cambridge, 1862	2			2
Clubionidae	*Clubiona lutescens* Westring, 1851	5		7	12
Clubionidae	*Clubiona reclusa* O. P.-Cambridge, 1863	2			2
Clubionidae	*Clubiona subsultans* Thorell, 1875	1	2		3
Cybaeidae	*Cryphoeca silvicola* (C. L. Koch, 1834)	1	8	1	10
Dictynidae	*Dictyna arundinacea* (Linnaeus, 1758)	13			13
Dictynidae	*Dictyna pusilla* Thorell, 1856		1		1
Gnaphosidae	*Drassodes pubescens* (Thorell, 1856)	2			2
Gnaphosidae	*Drassyllus pusillus* (C. L. Koch, 1833)	4			4
Gnaphosidae	*Micaria pulicaria* (Sundevall, 1831)	4			4
Gnaphosidae	*Zelotes clivicola* (L. Koch, 1870)	1			1
Linyphiidae	*Agyneta affinis* (Kulczynski, 1898)	17			17
Linyphiidae	*Agyneta cauta* (O. P.-Cambridge, 1903)	2			2
Linyphiidae	*Agyneta conigera* (O. P.-Cambridge, 1863)			2	2
Linyphiidae	*Agyneta ramosa* Jackson, 1912		7	13	20
Linyphiidae	*Agyneta subtilis* (O. P.-Cambridge, 1863)		1		1
Linyphiidae	*Bathyphantes parvulus* (Westring, 1851)	19			19
Linyphiidae	*Centromerus arcanus* (O. P.-Cambridge, 1873)		11	19	30
Linyphiidae	*Centromerus incilium* (L. Koch, 1881)	1			1
Linyphiidae	*Ceratinella brevis* (Wider, 1834)			1	1
Linyphiidae	*Ceratinella scabrosa* (O. P.-Cambridge, 1871)	59			59
Linyphiidae	*Dicymbium nigrum* (Blackwall, 1834)	18			18
Linyphiidae	*Dicymbium tibiale* (Blackwall, 1836)		3	3	6
Linyphiidae	*Diplocephalus latifrons* (O. P.-Cambridge, 1863)		2	7	9
Linyphiidae	*Diplocephalus picinus* (Blackwall, 1841)			11	11
Linyphiidae	*Diplostyla concolor* (Wider, 1834)	8	31	27	66
Linyphiidae	*Dismodicus bifrons* (Blackwall, 1841)		1	1	2
Linyphiidae	*Dismodicus elevatus* (C. L. Koch, 1838)			1	1
Linyphiidae	*Entelecara erythropus* (Westring, 1851)			5	5
Linyphiidae	*Erigone atra Blackwall*, 1833			1	1
Linyphiidae	*Erigonella hiemalis* (Blackwall, 1841)	2			2
Linyphiidae	*Gongylidiellum murcidum* Simon, 1884	9			9
Linyphiidae	*Gongylidium rufipes* (Linnaeus, 1758)	1	3	87	91
Linyphiidae	*Hypomma cornutum* (Blackwall, 1833)			1	1
Linyphiidae	*Incestophantes kochiellus* (Strand, 1900)		2		2
Linyphiidae	*Kaestneria pullata* (O. P.-Cambridge, 1863)	1			1
Linyphiidae	*Macrargus rufus* (Wider, 1834)			2	2
Linyphiidae	*Maso sundevalli* (Westring, 1851)			1	1
Linyphiidae	*Micrargus herbigradus* (Blackwall, 1854)	1			1
Linyphiidae	*Microlinyphia pusilla* (Sundevall, 1830)	9			9
Linyphiidae	*Microneta viaria* (Blackwall, 1841)		3	2	5
Linyphiidae	*Minicia marginella* (Wider, 1834)	1			1
Linyphiidae	*Neriene montana* (Clerck, 1757)		3	1	4
Linyphiidae	*Neriene peltata* (Wider, 1834)	1	21	101	123
Linyphiidae	*Nusoncus nasutus* (Schenkel, 1925)	1	5		6
Linyphiidae	*Obscuriphantes obscurus* (Blackwall, 1841)		5	4	9
Linyphiidae	*Oedothorax gibbosus* (Blackwall, 1841)	52		4	56
Linyphiidae	*Oryphantes angulatus* (O. P.-Cambridge, 1881)	3			3
Linyphiidae	*Palliduphantes pallidus* (O. P.-Cambridge, 1871)		1		1
Linyphiidae	*Pelecopsis elongata* (Wider, 1834)			1	1
Linyphiidae	*Pityohyphantes phrygianus* (C. L. Koch, 1836)		4	4	8
Linyphiidae	*Pocadicnemis pumila* (Blackwall, 1841)	43			43
Linyphiidae	*Poeciloneta variegata* (Blackwall, 1841)		1		1
Linyphiidae	*Porrhomma campbelli* F. O. P.-Cambridge, 1894			1	1
Linyphiidae	*Porrhomma pallidum* Jackson, 1913	1	2		3
Linyphiidae	*Porrhomma pygmaeum* (Blackwall, 1834)	1			1
Linyphiidae	*Tapinocyba insecta* (L. Koch, 1869)	1			1
Linyphiidae	*Tapinocyba pallens* (O. P.-Cambridge, 1873)	1			1
Linyphiidae	*Tenuiphantes alacris* (Blackwall, 1853)		5	4	9
Linyphiidae	*Tenuiphantes tenebricola* (Wider, 1834)		10	22	32
Linyphiidae	*Thyreostenius parasiticus* Westring, 1851		1		1
Linyphiidae	*Tiso vagans* (Blackwall, 1834)	8		1	9
Linyphiidae	*Walckenaeria antica* (Wider, 1834)	5			5
Linyphiidae	*Walckenaeria atrotibialis* (O. P.-Cambridge, 1878)	6	1	15	22
Linyphiidae	*Walckenaeria dysderoides* (Wider, 1834)	1	1		2
Linyphiidae	*Walckenaeria kochi* (O. P.-Cambridge, 1873)	1			1
Linyphiidae	*Walckenaeria obtusa* Blackwall, 1836			1	1
Linyphiidae	*Walckenaeria unicornis* O. P.-Cambridge, 1861	1			1
Linyphiidae	*Walckenaeria vigilax* (Blackwall, 1853)	15			15
Liocranidae	*Agroeca brunnea* (Blackwall, 1833)	2			2
Lycosidae	*Alopecosa pulverulenta* (Clerck, 1757)	14			14
Lycosidae	*Pardosa amentata* (Clerck, 1757)	1			1
Lycosidae	*Pardosa fulvipes* (Collett, 1876)	362			362
Lycosidae	*Pardosa lugubris* (Walckenaer, 1802)	17			17
Lycosidae	*Pardosa paludicola* (Clerck, 1757)	2			2
Lycosidae	*Pardosa palustris* (Linnaeus, 1758)	1			1
Lycosidae	*Pardosa riparia* (C. L. Koch, 1833)	290			290
Lycosidae	*Pardosa sphagnicola* (Dahl, 1908)	2			2
Lycosidae	*Piratula hygrophila* (Thorell, 1872)	10		4	14
Lycosidae	*Trochosa terricola* Thorell, 1856		1		1
Lycosidae	*Xerolycosa miniata* (C. L. Koch, 1834)	1			1
Miturgidae	*Zora armillata* Simon, 1878	1			1
Miturgidae	*Zora spinimana* (Sundevall, 1833)	2			2
Oxyopidae	*Oxyopes ramosus* (Martini & Goeze, 1778)	1			1
Philodromidae	*Philodromus emarginatus* (Schrank, 1803)	1			1
Philodromidae	*Tibellus oblongus* (Walckenaer, 1802)	28			28
Phrurolithidae	*Phrurolithus festivus* (C. L. Koch, 1835)	2			2
Salticidae	*Evarcha arcuata* (Clerck, 1757)	1			1
Salticidae	*Evarcha falcata* (Clerck, 1757)	1			1
Salticidae	*Heliophanus flavipes* (Hahn, 1832)	1			1
Sparassidae	*Micrommata virescens* (Clerck, 1757)	2			2
Tetragnathidae	*Metellina mengei* (Blackwall, 1869)	2	43	49	94
Tetragnathidae	*Pachygnatha degeeri* Sundevall, 1830	4			4
Tetragnathidae	*Pachygnatha listeri* Sundevall, 1830			2	2
Tetragnathidae	*Tetragnatha pinicola* L. Koch, 1870	1		1	2
Theridiidae	*Episinus angulatus* (Blackwall, 1836)	1			1
Theridiidae	*Euryopis flavomaculata* (C. L. Koch, 1836)	19			19
Theridiidae	*Lasaeola tristis* (Hahn, 1833)	1			1
Theridiidae	*Neottiura bimaculata* (Linnaeus, 1767)	13		1	14
Theridiidae	*Phylloneta impressa* (L. Koch, 1881)	11			11
Theridiidae	*Robertus lividus* (Blackwall, 1836)	4	8	3	15
Theridiidae	*Robertus neglectus* (O. P.-Cambridge, 1871)	1		3	4
Theridiidae	*Theridion mystaceum* L. Koch, 1870		1	1	2
Theridiidae	*Theridion varians* Hahn, 1833		2	7	9
Theridiidae	*Thymoites bellissimus* (L. Koch, 1879)			1	1
Theridiidae	*Yunohamella palmgreni* (Marusik & Tsellarius, 1986)		16		16
Thomisidae	*Misumena vatia* (Clerck, 1757)	1			1
Thomisidae	*Ozyptila praticola* (C. L. Koch, 1837)			2	2
Thomisidae	*Ozyptila trux* (Blackwall, 1846)	63	1	7	71
Thomisidae	*Xysticus audax* (Schrank, 1803)	1		1	2
Thomisidae	*Xysticus cristatus* (Clerck, 1757)	5			5
Thomisidae	*Xysticus lineatus* (Westring, 1851)	29		1	30
Thomisidae	*Xysticus ulmi* (Hahn, 1831)	85			85
**Individuals**	**1307**	**214**	**434**	**1956**
**Species richness**	**78**	**34**	**45**	**115**
